# Dependence of Gait Deviation on Weight-Bearing Asymmetry and Postural Instability in Children with Unilateral Cerebral Palsy

**DOI:** 10.1371/journal.pone.0165583

**Published:** 2016-10-27

**Authors:** Małgorzata Domagalska-Szopa, Andrzej Szopa, Andrzej Czamara

**Affiliations:** 1 Department of Medical Rehabilitation, School of Health Sciences in Katowice, Medical University of Silesia, Katowice, Poland; 2 Department of Physiotherapy, School of Health Sciences in Katowice, Medical University of Silesia, Katowice, Poland; 3 College of Physiotherapy in Wrocław, Wrocław, Poland; Rutgers University Newark, UNITED STATES

## Abstract

Postural control deficits have been suggested to be a major component of gait disorders in children with cerebral palsy. The purpose of this study was to investigate the relationship between postural stability and treadmill walking, in children with unilateral cerebral palsy, by defining dependence between the posturographic weight-bearing distribution and center of pressure (CoP) sway during quiet standing with Gillette Gait Index and the 16 distinct gait parameters that composed the Gillette Gait Index. Forty-five children with unilateral cerebral palsy from 7–12 years of age were included in this study. A posturographic procedure and 3-dimensional instrumented gait analysis was developed. In general, across the entire tested group, the significant correlations concerned only the asymmetry of the weight bearing and a few of the distinct gait parameters that compose the Gillette Gait Index; moreover, correlation coefficients were low. The division of subjects into two clinical subgroups: children that exhibited a tendency to overload (1) and to underload (2) the affected body side, modified the results of the explored relationships. Our findings revealed that the difficulties experienced by children with hemiplegia while controlled in a standing position result from tendency to excessively or insufficiently load the affected lower limbs, and thus establishes a direct relationship with inadequate affected peak ankle DF in both stance and swing gait phases. Given the presented relationship between postural instability and deviation of the particular gait parameters in children with unilateral cerebral palsy, a follow-up study will be needed to determine the therapeutic approaches that will be most effective in promoting increased improvement in gait pattern, as well as the static and dynamic balance in standing.

## Introduction

Postural control ensures the proper positioning of the body in space and maintains stability and body alignment by keeping the projection of the center of pressure (CoP) within the limits of the support base [1]. In the case of cerebral palsy (CP), postural controls are impaired according to the site and extent of brain damage [2–8].

Postural control deficits have been suggested to be a major component of gait disorders in CP [6,9–12]; however, the relationship between standing balance and walking function has rarely been investigated. Although the literature has highlighted the relationship between postural control and functionality, there is limited information regarding the relationship between postural control and walking, and it is not clear which parameter of standing balance correlates with which gait parameter.

The present study is a follow-up to a three-part series on the functional assessment of children with unilateral CP, which included the following: 1) postural pattern analysis, 2) gait analysis, and 3) postural stability [13–16].

In our previous studies on the weight-bearing (WB) distribution between the affected and unaffected body sides, two different postural patterns were described: the progravitational postural pattern, characteristic for children with tendency to overload the affected body side, and an antigravitational postural pattern characteristic for children with a tendency to underload the affected body side [13,16]. In the following studies based on measuring WB between the sides in children with unilateral CP, we found that children who underloaded the affected body side had poorer postural stability and a higher degree of deviation from normal gait than those who overloaded the affected side [14,15]. Accordingly, we decided to test whether the relationship between postural stability and walking for children with unilateral CP is dependent on weight distribution between the affected and unaffected body sides.

The primary aim of this investigation was to assess the relationship between postural instability while standing and gait deviation in children with unilateral CP during treadmill walking. Specifically, the correlations between the WB distribution and the CoP shifts during quiet standing and the selected gait parameters that composed the Gillette Gait Index (GGI) were studied. It was hypothesized that a tendency to overload or underload the affected body side would predict not only particular disturbances of postural control but also specific gait disturbances.

## Materials and Methods

The study was approved by The Ethical Committee of Medical University of Silesia and conformed to the declaration of Helsinki. All the patients and their parents/guardians provided written informed consent prior to the study, including enrolment and data collection.

### Participants

The study group consisted of 45 children with unilateral CP, classified as Level I and Level II, based on the Gross Motor Classification System (GMFCS) and Type 1–4 according to classification of hemiplegia gait [17,18]. Each participant met all of the following inclusion criteria: a diagnosis of unilateral CP; age over 7 years (to minimize the incidence of unstable gait patterns); ability to walk without assistance; and ability to follow verbal directions. All subjects were attending the outpatients department at a local center for physiotherapy interventions. The subjects are described in Table 1.

**Table 1:** Demographic characteristics of children with unilateral CP.

		TOTAL	ULA	OLA
	Age	9.8±2.1	10.2±2.2	9.4±1.9
Gender	Girls [n]	17	9	8
	Boys [n]	28	13	15
Side	Right [n]	29	14	15
	Left [n]	16	8	8
GMFM	I [n]	34	17	18
	II [n]	11	5	6
Type of hemiplegia gait	Type 1 [n]	8	5	3
	Type 2A [n]	13	-	13
	Type 2B [n]	7	-	7
	Type 3 [n]	9	9	-
	Type 4 [n]	8	8	-

Number of subjects in gender, hemiplegic side, level GMFM [17] and classification of hemiplegia gait according to Rodda [18]; TOTAL, children with unilateral and in two clinical subgroups; ULA, children with unloading of the affected body side; OLA, children with overloading of the affected side.

The exclusion criteria were as follows: (1) receiving pharmacological agents at the time of the study; (2) spasticity management during the 6 months before the evaluation; (3) previous hip dislocation or fracture of the lower limbs; (4) a history of uncontrolled seizures or vestibular dysfunction; and (5) any accompanying disease that could influence the gait pattern (e.g., cardiopulmonary disorders, diabetes, or asthma).

The reference group consisted of 25 typically-developing children (15 girls and 10 boys), with a mean age of 8 years and 8 months (range: 7 years and 5 months to 12 years and 3 months). These children underwent the posturographic WB distribution and 3DGA for the purposes of defining the Index of Asymmetry (AI) of weight distribution between body sides and GGI calculation.

### Methods

The examination consisted of three interrelated parts: (1) posturographic WB distribution; (2) posturographic (PG) testing (CoP measurements), and (3) 3DGA.

During posturography the subjects stood comfortably with open eyes and with their arms alongside their trunk on the platform, barefoot, in a quiet stance position. The distance between their parallel heels was approximately 3 cm (feet abducted at 20°). First, the WB distribution and then posturographic CoP using a force platform (PDM Multifunction Force Measuring Plate, Zebris, Germany) were measured. The duration of each measurement was 30 s. Each measurement was recorded three times (3 trials, each lasting for 30 s, with a 30 s pause between trials). The mean values from three trials were used for future analysis.

3DGA was performed using the Compact Measuring System for 3D Real–Time Motion Analysis (CMS–HS 3D) based on 15 active ultrasonic markers (5 triplicate ultrasound markers) with WinGait software (Zebris Medizintechnik GmbH, Germany) [14,15]. The gait data were recorded as the subjects walked without shoes and without assistive devices on a treadmill (Alfa XL, Kettler, Germany). Each child’s typical over-ground walking speed (spontaneous) and time taken to walk 10 m were collected by a single examiner before the gait analysis. Prior to data collection, all of the subjects were given the opportunity to practice walking on the treadmill. Based on the spontaneous speed of walking, treadmill belt speeds were calculated as values in kilometers per hour. Depending on each subject’s walking abilities, five to eight gait cycles were recorded. Separate measurements for the unaffected and affected gait cycles for each subject were obtained. An experienced physical therapist selected the six gait cycles per subject for further analysis, which were the most characteristic of the child in his opinion. The kinematic data were averaged from three randomly selected cycles of these six. The data collected were reported using WinGait software.

Additionally, the limb lengths were measured using a tape measure method (TMM). TMM measurements were obtained by one person. During measurement, subjects were positioned supine on a settee and their lower extremities were exposed. Both lower limbs were placed as closely as possible to the anatomical position. During measurement the examiner placed one end of the tape measure on the anterior superior iliac spine (ASIS) and then gradually guided the tape down the anteromedial aspect of the subject's thigh, patella, and lower leg to the medial malleolus. The examiner then held the tape taut and another person recorded the value from the opposite surface of the tape. The values were rounded to the nearest 0.5 cm.

### Data analysis

The WB asymmetry, as the difference of the WB values of the right from those of the left body sides for reference group, was calculated. The standard deviation of WB asymmetry, which was 9.83% was considered as the Index of Asymmetry (AI) [16]. The WB asymmetry between affected and unaffected body sides was calculated for children with CP. This value of AI in references was used as a criterion to define the asymmetry of WB on the affected/unaffected body sides in children with CP and for the selection of two clinical subgroups: 1) ULA–children who underloaded the affected body side (AI > 9.83%); and 2) OLA–children who overloaded of the affected side (AI < 9.83%)” [16].

Additionally, differences between the length of the affected and unaffected lower limbs within and between clinical subgroups (ULA; OLA) were calculated. These were not statistically different within clinical subgroups (66.5±11.0 cm, 66.0±11.0 cm, p = 0.909; 68.0±7.0 cm, 68.0±7.0, p = 0.869; respectively); and between clinical subgroups (66.5±11.0 cm, 68.0±7.0 cm, p = 0.603; 66.0±11.0 cm, 68.0±7.0 cm, p = 0.431; respectively).

The WB distribution, the CoP shifts and surface area of the CoP were calculated (Table 2). To characterize the gait patterns, the GGI was used, with the procedure described by Schutte et al. [19]. The GGI was calculated, separately, for affected and unaffected lower limbs based on the 16 selected gait parameters taken from the objective gait analysis data, including the 1) stance phase, expressed as the percentage of the gait cycle; 2) walking speed, normalized to leg length; 3) cadence; 4) mean pelvic tilt; 5) ROM of pelvic tilt; 6) mean pelvic rotation; 7) minimum hip flexion; 8) ROM of hip flexion/extension; 9) peak hip abduction in swing; 10) mean hip rotation in stance; 11) knee flexion at initial contact; 12) time to peak knee flexion in swing, expressed as the percentage of the gait cycle; 13) ROM of knee flexion; 14) peak dorsiflexion in stance; 15) peak dorsiflexion in swing; and 16) mean foot progression [19,20].

**Table 2:** Posturographic weight-bearing distribution and posturographic CoP shifts and surface area of the CoP.

WB distribution	QA [%]	Affected foot percentage load distribution
CoP shifts	SP [cm]	Sway path length of the CoP (SP)
	SDx [cm]	Standard deviation of x'
	SDy [cm]	Standard deviation of y'
Surface area of the CoP	WoE [cm]	Width of the ellipse (medial-lateral sway path of the CoP)
	HoE [cm]	Height of the ellipse (anterior-posterior sway path of the CoP)
	AoE [cm^2^]	Area of the centers of pressure (calculated from the CoP shifts in such a way that 95% of the data are within the ellipsoid and 5% are outside).

GGI is a numerical calculation of how close a given gait pattern is to a normal gait pattern typical of children without disabilities. GGI uses principal component analysis on a set of 16 independent kinematic variables that describe a subject’s gait [19]. The strength of the GGI is that it is based on the most important spatiotemporal variables and kinematic parameters from 3–dimensional gait analysis (3DGA). For the GGI project, a group of experienced clinicians were polled to arrive at an appropriate set of variables that they felt correlated closely with particular gait problems [19]. Moreover, GGI was shown to be efficient in categorizing pathology, to be clinically applicable [21], and most importantly, GGI correlated well with physical functioning [22].

The intraclass correlation coeficient (ICC 2.1) with 95% confidence interval to measure the intra-observer agreement was calculated for the gait analysis (GGI) based on two examinations performed by the same assessor of 10 subjects. GGI demonstrated good to high level of intra-observer repeatability, with the ICC ranging from 0.72 to 0.91. The between–trial repeatability of the PG measurement (ICC 1.3) was evaluated based on two examinations of 10 subjects. All of the outcomes from the PG demonstrated medium to good repeatability, with the ICC ranging from 0.58 to 0.72. The repeatability was assessed based on the interpretation of ICC (similar to the interpretation of the correlation coefficient): 0–0.2 –very low, 0.21–0.4 –low, 0.41–0.6 –medium, 0.61–0.8 –good, and 0.8–1 –high repeatability.

### Statistical analysis

The normality of the distribution of the analysed parameters was assessed using the skewness and kurtosis and the Shapiro–Wilk test. The Spearman Rank Correlation test was used to examine the relationship between the posturographic WB distribution, posturographic CoP shifts, and GGI as well as the specific 16 gait parameters that composed the GGI. The correlations were performed for the whole tested group, and separately for clinical subgroups. Coefficients with a p–value of less than 0.05 were considered significant. The correlations were interpreted according to the guidelines adopted from Altman [23]: Rs<0.2, poor; 0.21–0.4, fair; 0.41–0.6, moderate; 0.61–0.8, good; and 0.81–1, very good. The software package Statistica (version 10.0 PL) was used for statistical analysis.

## Results

A summary of the WB distributions, posturographic CoP shifts, and surface area of the CoP, for all of the tested and clinical subgroups is presented in Table 3. The descriptive statistics of the GGI and the 16 distinct gait datasets that compose the GGI are presented in Table 4. Table 5 summarizes the significant correlation coefficients (p–value < 0.05) between the WB distribution, CoP measurements, and GGI, in addition to the 16 distinct gait datasets that compose the GGI of the affected lower limbs of all of the tested and clinical subgroups.

**Table 3:** Summary of the posturographic weight-bearing distribution and posturographic CoP shifts and surface area of the CoP (as shown in Table 2).

		TOTAL (n = 45)	ULA (n = 22)	OLA (n = 23)
WB distribution	QA [%]	49.2±4.9	53.4±14.2	48.7±14.5
CoP shifts	SDx [cm]	0.6±0.2	0.6±0.2	0.6±0.2
	SDy [cm]	0.6±0.2	0.6±0.2	0.5±0.2
	SP [cm]	113.9±3.3	119.9±35.4	108.2±23.9
Surface area of the CoP	WoE [cm]	5.1±3.3	5.7±3.8	3.1±1.8
	HoE [cm]	4.4±2.4	4.1±2.0	4.4±2.5
	AoE [cm^2^]	15.9±10.0	15.9±8.7	15.8±11.2

TOTAL, children with unilateral CP and in two clinical subgroups; ULA, children with underloading of the affected body side; OLA, children with overloading of the affected side.

**Table 4:** Summary of the GGI values and 16 distinct gait datasets that compose the GGI for affected lower limbs. Normally distributed variables are summarised as the means and standard deviations, and non-normally distributed variables are presented as the median and range.

Affected lower limb	TOTAL (n = 45)	ULA (n = 22)	OLA (n = 23)
GGI	92.2±58.5	113.8±65.0	71.6±43.7
1. Time of toe off [% gait cycle]	61<49˗73>	59<47˗76>	58.5<50˗69>
2. Walking speed/leg length [km/h]	0.5±0.1	0.5±0.1	0.5±0.1
3. Cadence [step/sec]	0.7±0.1	0.8<0.4˗1.1>	0.7<0.4˗0.9>
4. Mean pelvic tilt [°]	12.8±4.8	13.9±5.8	11.1<6.3˗19.7>
5. ROM of pelvic tilt [°]	8.2±3.0	8.2±3.0	8.1<3.2˗7.9>
6. Mean pelvic rotation [°]	-1.7±7.5	-8.5±4.5	5.2<-1.6˗11.1>
7. Minimum hip flexion [°]	-4.9<-37.9˗23.2>	-12.0<-37.9˗18.2>	5.2±9.7
8. ROM of hip flex/ext [°]	27.7<12.5˗53.8>	25.3±8.7	31.3<21.8˗45.3>
9. Peak hip abduction in SW [°]	4.2<-25.5˗32.5>	-11.0<-25.5˗0.2>	11.8±5.7
10. Mean hip rotation in ST [°]	14.4±8.1	16.7±10.3	13.0<4.3˗20.4>
11. Knee flexion at IC [°]	14.3±11.3	4.9<0.9˗14.1>	22.8±9.6
12. Time of peak knee flex in SW [%]	54.3<17.0˗82.4>	45.3±10.3	62.4<54.1˗82.4>
13. ROM of knee flexion[°]	46.3<25.4˗66.7>	40.4<27.8˗59.6>	52.1<25.4˗66.7>
14. Peak dorsiflexion in ST [°]	8.4±10.1	-1.8<-5.8˗3.9>	17.6±4.1
15. Peak dorsiflexion in SW [°]	1.9<-8.7˗16.1>	-1.8<-8.7˗2.6>	8.1<0.8˗16.1>
16. Mean foot progression [°]	-3.9<-30.6˗24.0>	-0.7<-30.6˗24.0>	-6.34<-27.3˗20.4>
Step length [m]	0.3±0.1	0.3±0.1	0.3<0.2˗0.4>

TOTAL, children with unilateral CP and in two clinical subgroups; ULA, children which underloading of the affected body side; OLA, children which overloading of the affected side. The normally distributed variables were summarized by means and standard deviations; non-normally with median and range.

**Table 5:** Spearman’s correlation coefficients for the relationships of the posturographic weight-bearing distribution and the posturographic CoP shifts and surface area of the CoP (as shown in Table 1) with the Gillette Gait Index and 16 distinct gait parameters that composed the GGI for the affected lower limbs.

	QA [%]			SP [cm]			SDx [cm]			SDy [cm]			HoE [cm]			WoE [cm]			AoE [cm^2^]		
affected lower limb	TOTAL	ULA	OLA	TOTAL	ULA	OLA	TOTAL	ULA	OLA	TOTAL	ULA	OLA	TOTAL	ULA	OLA	TOTAL	ULA	OLA	TOTAL	ULA	OLA
GGI	-	-0.64	0.46*	˗	˗	˗	˗	˗	˗	˗	˗	˗	˗	˗	˗	˗	˗	˗	˗	˗	˗
1. Time of toe off [% gait cycle]	˗	˗	˗	˗	˗	˗	˗	˗	˗	˗	˗	˗	˗	˗	˗	˗	˗	˗	˗	˗	˗
2. Walking speed/leg length [km/h]	˗	˗	˗	˗	˗	˗	˗	˗	˗	˗	˗	˗	˗	˗	˗	˗	˗	˗	˗	˗	˗
3. Cadence [step/sec]	˗	˗	˗	˗	˗	˗	˗	˗	˗	˗	˗	˗	˗	˗	˗	˗	˗	˗	˗	˗	˗
4. Mean pelvic tilt [°]	˗	˗	˗	˗	˗	˗	˗	˗	˗	˗	˗	˗	˗	˗	˗	˗	˗	˗	˗	˗	˗
5. ROM of pelvic tilt [°]	˗	˗	˗	˗	˗	0.52*	˗	-0.59*	-0.47*	˗	0.55	˗	˗	-0.61*	˗	0.35*	˗	˗	˗	˗	˗
6. Mean pelvic rotation [°]	˗	˗	˗	˗	˗	˗	˗	˗	-0.46*	˗	˗	˗	˗	˗	˗	˗	0.41	˗	˗	˗	˗
7. Minimum hip flexion [°]	˗	˗	˗	˗	˗	˗	˗	˗	˗	˗	˗	˗	˗	˗	˗	˗	˗	˗	˗	˗	˗
8. ROM of hip flex/ext [°]	0.36*	˗	˗	˗	˗	˗	˗	˗	˗	˗	˗	˗	˗	˗	˗	˗	˗	˗	˗	˗	˗
9. Peak hip abduction in SW [°]	0.38**	˗	˗	˗	˗	˗	˗	˗	˗	˗	˗	˗	˗	˗	˗	˗	˗	˗	˗	˗	˗
10. Mean hip rotation in ST [°]	˗	˗	˗	˗	-0.45	˗	˗	˗	˗	˗	0.42	˗	˗	-0.51*	˗	˗	˗	˗	˗	˗	˗
11. Knee flexion at IC [°]	˗	˗	˗	˗	˗	˗	˗	˗	˗	˗	˗	˗	˗	˗	˗	˗	˗	˗	˗	˗	˗
12. Time of peak knee flex in SW [%]	˗	˗	˗	˗	˗	˗	˗	˗	˗	˗	˗	˗	˗	˗	˗	˗	˗	˗	˗	˗	˗
13. ROM of knee flexion[°]	0.32	0.48	˗	˗	˗	˗	˗	˗	˗	˗	˗	˗	˗	˗	0.53*	˗	˗	˗	˗	˗	˗
14. Peak dorsiflexion in ST [°]	0.38**	˗	0.65**	˗	-0.75*	0.49*	˗	˗	˗	˗	˗	˗	˗	˗	˗	˗	˗	0.62*	˗	˗	0.55*
15. Peak dorsiflexion in SW [°]	0.50**	˗	˗	˗	-0.63	0,45*	˗	0.45	˗	˗	˗	˗	˗	0.43	˗	˗	˗	˗	˗	0.44	˗
16. Mean foot progression [°]	˗	˗	-0.61**	˗	˗	˗	˗	˗	˗	˗	˗	˗	˗	˗	˗	˗	˗	˗	˗	˗	˗
Step length [m]	˗	0.68*	˗	˗	˗	˗	˗	˗	˗	˗	˗	˗	˗	˗	˗	˗	˗	˗	˗	˗	˗

TOTAL, children with unilateral CP and in two clinical subgroups; ULA, children with underloading of the affected body side; OLA, children with overloading of the affected side.

p < 0.05

*, p < 0.01

**, p < 0.001

Across the entire tested group, no significant correlations of the WB distributions, or the CoP shifts with the GGI, or the gait data comprising the GGI, were found. In general, across the entire tested group, the significant correlations concerned only the asymmetry of the WB and a few of the distinct gait parameters that composed the GGI; moreover, Spearman’s correlation coefficients (Rs) were low.

The division of subjects into clinical subgroups revealed the greatest number and strengths of significant correlations. The correlations were predominantly moderate to good (Table 5). Significant correlations between the WB and GGI were noted. A strong negative correlation for the underloaded affected limb and a moderate positive correlation for the overloaded limb were noted (Rs = -0.61, p < 0.001 and Rs = 0.46, p < 0.001, respectively). Although none of the correlations between the CoP measurement and GGI were statistically significant, further statistical analysis revealed significant correlations of the CoP measurement with the gait data, but these relationships were different for the underloaded and overloaded affected lower limbs. The sway path length of the CoP (SP) was correlated with the peak dorsiflexion (DF) of the affected lower limb of both the stance and swing phases, but reverse correlations were noted: negatively with underloaded (Rs = -0.75, p < 0.01 and Rs = -0.63, p < 0.05, respectively) and positively with the overloaded one (Rs = 0.49, p < 0.01 and Rs = 0.45, p < 0.01, respectively) (Table 5). The area of the CoP correlated with the peak DF of both types of affected lower limb although different relationships were observed: a strong correlation between peak DF of the overloading leg in stance phase and medial-lateral sway of the CoP (WoE) (Rs = 0.62, p < 0.05), and good correlation between peak DF of the underloaded limb in swing with the anterior-posterior sway of the CoP (HoE) (Rs = 0.43, p < 0.05). Additionally, the anterior-posterior sway of the CoP (HoE) was strongly negatively correlated with the pelvic ROM in the sagittal planes (Rs = -0.61, p < 0.01) and with hip rotation in stance of the underloaded affected lower limb (Rs = -0.51, p < 0.01) (Table 5).

## Discussion

The results of the present study only partially confirmed the dependence of gait disturbances on postural instability in children with unilateral CP. Although the present study detected a WB asymmetry in the limited data from children with unilateral CP, in general, the r-values were low. Moreover, no associations of CoP displacement with the degrees of the deviations from the normal gait (GGI) or the distinct gait data comprising the GGI were noted (Table 5). These findings may be partly attributable to the fact that the group of children with unilateral CP appeared to be relatively homogeneous, but their postural stabilities and gait patterns differed depending on their tendencies to underload or overload the affected body side, as we have previously reported [13–16]. The present study confirmed that children with a tendency to underload the affected body side exhibit greater asymmetry in WB, poorer stability control in standing (Table 3) and nearly two-fold greater GGI for the affected lower limb compared with children who tend to overload the affected body side (Table 4).

Exploration of the correlation between WB and gait that was separately performed according to clinical subgroups revealed that the WB asymmetry was strongly related to GGI and the distinct gait parameters that composed the GGI. We detected that the degree of gait deviation increased with the both the conditions of underloading and overloading of the affected side. Indeed, this association was much more strongly expressed by the children who underload the affected body side. However, this correlation also concerned the children with unilateral CP who overloaded the affected side of body. Thus far, it has been thought that children with unilateral CP tend to displace their weight only toward the unaffected body side [3,6,24]. This pattern may result from an improper approach to WB distribution measurements between the unaffected and affected sides of body in the population of children with unilateral CP. The average of the percentage load distribution toward the affected body side did not indicate any real WB asymmetry because the average from the negative (for children who underloaded the affected side) and positive values (for those who overload the affected side) was zero, which indicates a lack of asymmetry in this population or provides other results that depend on the particular “type” of the subject number. In this respect, our findings support our hypothesis that the relationship between postural stability and walking in children with unilateral CP can be differential and dependent on the weight distribution in terms of the affected and unaffected body sides. The division of the participants into two clinical subgroups according to the tendencies to underload or overload the affected body side allowed for a better understanding of the abovementioned relationship in the entire population of children with unilateral CP.

Further analyses of the dependence of the distinct gait parameters on postural stability revealed that among the children evaluated in the present study, changes in the body structure and function, which were represented by higher CoP oscillation values (indicative of less control over the postural adjustments required to maintain balance), correlated with the degree of DF of the affected ankle in both gait phases, although the reverse relationship within particular clinical groups was observed. The obtained results suggested that in children who overloaded the affected body side, the excessive DF of the affected ankle correlated with higher scores of insufficient displacement of the CoP (ML). In those who underloaded the affected body side, the insufficient DF of the affected ankle during walking correlated with higher scores of insufficient displacement of the CoP (AP) while standing. Additionally, in the last mentioned group of children the strong correaltions between greater pelvic tilt ROM and affected hip rotation during walking and lower AP displacement of the CoP were observed. This is consistent with the results of the gait analysis which revealed the fixed equines of affected leg during both the support and the swing gait phases in children who underloaded the affected body side. This can be explained by the preference for using the pelvis and hip compensatory strategy instead of an ankle strategy to control stability over the body. Children with CP present deficiencies in recruiting the muscles around the ankle joint and therefore preferably use muscles around the pelvis and hip, a strategy that is associated with lower AP displacements. The discrepancy between children who underloaded and those who overloaded the affected body side might result from different compensatory strategies for the standing posture that are possibly aimed to cope with pathophysiological factors, such as non-neural components (e.g., bony/joint deformities) and central motor deficits (e.g., paresis) [25].

The link between postural control and walking is obvious because of the common neurophysiological basis of the control of both types of behaviour [26]. Previous studies have reported the important relationship between static postural control while standing and the level of functional abilities in children with CP. The results showed that postural instability influences other domains of a child’s functional abilities and interferes with his/her activity levels in the domains of self-care and mobility [12,27]. Many studies found that the quality of walking depends on postural control in the standing position [3,4,11], or that one of the contributing factors to problems with the gait of CP children is poor postural control while standing [6–8,25,26,28]. To the best of our knowledge, the presented study is the first study recognizing specific dependence of gait deviation on postural instability. The division of a sample of children with unilateral CP into two clinical subgroups according to the tendencies to underload or overload the affected body side allowed us to define the specific correlation between WB distribution and the CoP oscillation in the standing position with deviation of particular gait parameters. As most of the available information on the relationship between postural stability and gait is derived from studies that have covered the whole population of children with CP, it is difficult to compare our results with those of other studies.

The results of the present study revealed that the difficulties experienced by children with hemiplegia while controlled in a standing position result from tendency to excessively or insufficiently load the affected lower limbs, and thus establishes a direct relationship with inadequate affected peak ankle DF in both stance and swing gait phases. It can be assumed that the tendency to overload or underload the affected body side would predict not only particular disturbances of postural control but also specific gait disturbances.

### Study Limitations

However, a couple of limitations need to be taken into account when interpreting our results. First, the findings from treadmill-based gait research can differ from overground gait data in children with CP. Despite, that many studies have investigated the differences between the treadmill and overground walking in children with CP, conclusive research has not been conducted to verify this assumption. Although, it is generally agreed that the treadmill walking can alter spatiotemporal gait variables, there still exist inconsistencies as to the kinematic differences between the two walking modalities [29,30,31]. Secondly, the present study was the failure to perform PG with the children’s eyes closed during the examination, which would add credence to these results. A portion of the subjects (approximately 23%) were not able to maintain a standing position with their eyes closed for 30 seconds, and the remaining subjects presented outcomes that exhibited very low repeatability.

## Conclusion

In recent years there is growing interest in partial body weight support (PBWS) as part of the treadmill training protocol for children with CP. The numerous studies indicate some positive benefits of PBWS not only in increase of the speed, endurance, and efficiency of overground walking [32,33], and other areas of gross motor function improvement for children with CP [34,35]. Our results suggest that the tendency to overload or underload the affected body side would predict particular disturbances of postural control and specific gait disturbances in children with unilateral CP. Therefore, we believe that is a potentially important clinical finding because this awareness may be essential in the programing of treadmill training with PBWS for children with hemiplegia. Given the presented relationship between postural instability and deviation of the particular gait parameters in children with unilateral CP, a follow-up study will be needed to determine the therapeutic approaches that will be most effective in promoting increased improvement in gait pattern, as well as the static and dynamic balance in standing.

## Abbreviations

3DGA: three-dimensional gait analysis
AI: Index of Asymmetry
AP: the anteroposterior amplitude of the CoP displacement
CoP: center of pressure
CP: cerebral palsy
DF: dorsiflexion
GGI: Gillette Gait Index
HoE: height of an ellipse
ML: the mediolateral amplitude of the CoP displacement
OLA: children who overloaded the affected side
PG: posturographic
ROM: range of motion
ULA: children who underloaded the affected body side
WB: weight bearing
WoE: width of an ellipse
